# Novel vectors expressing anti-apoptotic protein Bcl-2 to study cell death in Semliki Forest virus-infected cells

**DOI:** 10.1016/j.virusres.2007.08.008

**Published:** 2008-01

**Authors:** Kaja Kiiver, Andres Merits, Inga Sarand

**Affiliations:** a Estonian Biocentre, Riia Street 23, 51010 Tartu, Estonia; b Institute of Molecular and Cell Biology, University of Tartu, Nooruse 1, 50411 Tartu, Estonia

**Keywords:** Alphaviruses, Semliki Forest virus, Cell death, Bcl-2, AT3

## Abstract

Semliki Forest virus (SFV, *Alphavirus*) induce rapid shut down of host cell protein synthesis and apoptotic death of infected vertebrate cells. Data on alphavirus-induced apoptosis are controversial. In this study, the anti-apoptotic *bcl-2* gene was placed under the control of duplicated subgenomic promoter or different internal ribosome entry sites (IRES) and expressed using a novel bicistronic SFV vector. The use of IRES containing vectors resulted in high-level Bcl-2 synthesis during the early stages of infection. Nevertheless, in infected BHK-21 cells translational shutdown was almost complete by 6 h post-infection, which was similar to infection with appropriate control vectors. These results indicate that very early and high-level *bcl-2* expression did not have a protective effect against SFV induced shutdown of host cell translation. No apoptotic cells were detected at those time points for any SFV vectors. Furthermore, Bcl-2 expression did not protect BHK-21 or AT3-neo cells at later time points, and infection of BHK-21 or AT3-neo cells with SFV replicon vectors or with wild-type SFV4 did not lead to release of cytochrome *c* from mitochondria. Taken together, our data suggest that SFV induced death in BHK-21 or AT3-neo cells is not triggered by the intrinsic pathway of apoptosis.

## 1 Introduction

Semliki Forest virus (SFV) is a positive-stranded RNA virus in *Alphavirus* genus (family *Togaviridae*), a widely distributed group of human and animal pathogens (Strauss and Strauss, 1994). SFV genomic RNA (so-called 42S RNA) is approximately 11.5 kb long and encodes four non-structural proteins designated nsP1–4; these are involved in viral RNA synthesis. The remaining proteins form the virus capsid and envelope and are not essential for virus replication.

After virus entry, the 42S RNA is translated into a large non-structural polyprotein, which is processed to form an early and subsequently a late replicase complex (Strauss and Strauss, 1994). The early replicase mediates synthesis of the negative-stranded RNA complementary to the genomic 42S RNA. Minus strands are used by the late replicase as templates for the synthesis of new positive strand 42S RNA, and for transcription of subgenomic mRNAs encoding the structural proteins.

The structural genes of SFV are not required for replication and can be removed or replaced with a polylinker and/or with foreign gene sequences. This property forms the basis of the SFV-based replicon vector systems (Liljestrom and Garoff, 1991; Smerdou and Liljestrom, 1999). SFV-based replicon vectors mediate high-level expression of heterologous proteins. However, as with virus, vectors cause shutdown of cellular biosynthesis and induce apoptotic death (Glasgow et al., 1997, 1998; Scallan et al., 1997). This precludes long-term foreign gene expression, and several attempts to reduce the cytotoxicity of alphavirus vectors have been made (Fazakerley et al., 2002; Lundstrom et al., 2003, 2001; Perri et al., 2000).

The anti-apoptotic gene *bcl-2* is an antagonist of the intrinsic mitochondrial pathway of apoptosis (for reviews see Ashe and Berry, 2003; Cory and Adams, 2002; Tsujimoto and Shimizu, 2000). Bcl-2 can prevent release of cytochrome *c* from mitochondria, thus, precluding the apoptotic cascade (Kluck et al., 1997; Yang et al., 1997). Bcl-2 can block apoptosis induced by several viruses, including influenza virus and reovirus (Nencioni et al., 2003; Rodgers et al., 1997). Existing data on Bcl-2 in SFV- or Sindbis virus-induced apoptosis are contradictory. On one hand it has been shown that alphavirus-induced apoptosis of baby hamster kidney (BHK) cells, Chinese hamster ovary cells, rat insulinoma cells and rat prostatic adenocarcinoma (AT3) cells can be prevented by over-expression of Bcl-2 (Levine et al., 1993; Lundstrom et al., 1997; Mastrangelo et al., 2000; Scallan et al., 1997). Similarly, a Sindbis virus expressing Bcl-2 produces reduced encephalitis in infected mice (Levine et al., 1996). That Bcl-2 expression can block apoptosis, suggests involvement of intrinsic pathway of apoptosis. In contrast, other studies using rat embryo fibroblasts and monocyte cell lines overexpressing Bcl-2 failed to detect a protective effect against alphavirus-induced apoptosis (Grandgirard et al., 1998; Murphy et al., 2001).

The aim of this study was to determine whether expression of anti-apoptotic Bcl-2 directly from SFV-based replicon vectors in BHK-21 cells could be used to prolong co-expression of marker proteins from a bicistronic SFV replicon. Using the SFV1 vector system (Liljestrom and Garoff, 1991), the *bcl-2* gene was placed either under the control of a duplicated SFV subgenomic promoter or an internal ribosome entry site (IRES). It is possible that expression of Bcl-2 from the subgenomic promoter occurs too late to prevent cell death. Expression from an IRES element within the genomic RNA should be more rapid. We tested two different IRES elements, the Encephalomyocarditis virus IRES (EMCV-IRES) and the crucifer-infecting tobamovirus IRES (CR-IRES). The latter is a 148-nt element, which precedes the CR coat protein gene and displays IRES activity across all kingdoms (Dorokhov et al., 2002). Using this novel approach we demonstrate that early Bcl-2 expression does not protect SFV-infected BHK-21 cells from alphavirus-induced translational shutdown or cell death. Moreover, our results indicate that SFV-induced cell death in BHK-21 cells does not involve the release of cytochrome *c* from mitochondria, and most likely does not occur by the apoptotic intrinsic pathway.

## 2 Materials and methods

### 2.1 Plasmid construction

**Fig. 1:**
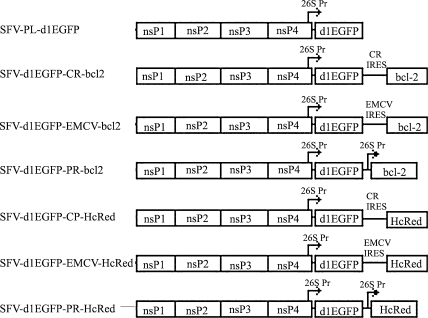
Schematic presentation of replicon vectors.

The BamHI-XmaI multicloning site of the pSFV1 replicon (Liljestrom and Garoff, 1991) was replaced with a BamHI, ApaI, ClaI, AvrII, NruI, NsiI and XmaI multicloning site; the resulting construct was designated as pSFV-PL. The spliced sequences encoding the mouse Bcl-2 alpha protein (locus AAA37282), the EMCV-IRES (pIRES2-EGFP; BD Clontech) and the 148 bp CR-IRES (Ivanov et al., 1997) were amplified by PCR, cloned and verified by sequence analysis. Each IRES was fused to the Bcl-2 coding sequence and cloned into NsiI-XmaI digested pSFV-PL vector; obtained constructs were designated as pSFV-EMCV-bcl2 and pSFV-CR-bcl2. To create constructs expressing Bcl-2 protein from the duplicated subgenomic promoter, the IRES from pSFV-EMCV-bcl2 was replaced by an oligonucleotide duplex representing the minimal SFV subgenomic promoter (Hertz and Huang, 1992); the resulting construct was designated pSFV-PR-bcl2. The d1EGFP reporter gene (BD Clontech) was amplified by PCR, sequenced and cloned into pSFV-PL, pSFV-EMCV-bcl2, pSFV-CR-bcl2 and pSFV-PR-bcl2 vectors treated with ClaI-NsiI. Resulting constructs were designated as pSFV-PL-d1EGFP, pSFV-d1EGFP-EMCV-bcl2, pSFV-d1EGFP-CR-bcl2 and pSFV-d1EGFP-PR-bcl2, respectively (Fig. 1). Sequences and primers are available upon request.

To construct SFV replicons expressing mutated chromoprotein HcRed (from the reef coral *Heteractis crispa*), *HcRed* was PCR amplified (from pHcRed1-N1; BD Clontech), cloned and sequenced. The sequence encoding Bcl-2 from pSFV-d1EGFP-EMCV-bcl2, pSFV-d1EGFP-CR-bcl2 and pSFV-d1EGFP-PR-bcl2 was replaced with *HcRed* to give constructs pSFV-d1EGFP-EMCV-HcRed, pSFV-d1EGFP-CR-HcRed and pSFV-d1EGFP-PR-HcRed (Fig. 1).

To obtain constructs used for viability analysis under puromycin selection, the sequence encoding d1EGFP from pSFV-PL-d1EGFP, pSFV-d1EGFP-EMCV-bcl2, pSFV-d1EGFP-CR-bcl2 and pSFV-d1EGFP-PR-bcl2 was replaced by that of puromycin acetyltransferase (*Pac*), and constructs were designated pSFV-PL-Pac, pSFV-Pac-EMCV-bcl2, pSFV-Pac-CR-bcl2 and pSFV-Pac-PR-bcl2.

To generate infectious RNA, constructs were linearised by SpeI digestion and in vitro transcription was carried out as previously described (Karlsson and Liljestrom, 2003).

### 2.2 Cells and viruses

BHK-21 cells were grown in Glasgow's Minimal Essential Medium containing 5% foetal calf serum, 0.3% tryptose phosphate broth, 0.1 U/ml penicillin and 0.1 μg/ml streptomycin. AT3-neo and AT3-bcl2 cells were grown in Roswell Park Memorial Institute-1640 medium containing 10% foetal calf serum, 0.1 U/ml penicillin and 0.1 μg/ml streptomycin. All cells were grown at 37 °C in a 5% CO_2_ atmosphere. SFV4 was derived from the infectious cDNA clone pSP6-SFV4 (Liljestrom et al., 1991).

### 2.3 Transfection and collection of virus-like particles (VLPs)

BHK-21 cells were co-transfected with equal amounts of vector and helper RNA (Liljestrom and Garoff, 1991). Helper RNA encodes the structural proteins under subgenomic promoter. Transfected cells were grown at 28 °C for 72 h and the VLPs collected, concentrated, purified and titrated as described by Karlsson and Liljestrom (2003). Although the replicase encoded by the replicon vector will amplify both RNAs, helper RNA is not packed into VLPs due to a missing packaging signal. To determine if any replication-proficient viruses were formed due to the recombination between replicon vector RNA and helper RNA, batches of VLPs were tested as described by Smerdou and Liljestrom (1999). All infections with VLPs were carried out in BHK-21 cells at a multiplicity of infections 10 (moi = 10) for 1 h at 37 °C.

### 2.4 Metabolic labelling

BHK-21 cells in 35 mm diameter plates were infected with SFV VLPs as described above. Infected cells were washed twice with PBS, once with methionine and cysteine free Dulbecco's Modified Eagle Medium followed by 30 min labelling with 50 μCi/ml of [^35^S]methionine and [^35^S]cysteine (RedivuePRO-MIX, Amersham Biosciences). After labelling, cells were washed with PBS, lysed in Laemmli buffer and analyzed by SDS-PAGE. Gels were dried under vacuum and exposed to film.

### 2.5 Immunoblot analysis

BHK-21 cells were infected and samples collected as described above. After SDS-PAGE, proteins were transferred to a nitrocellulose membrane, probed with rabbit polyclonal antisera against SFV nsP1, EGFP (in-house), HcRed (Clontech), with a mouse monoclonal antibody against Bcl-2 (Santa Cruz Biotechnology, Inc.) or with a mouse monoclonal antibody against beta-actin (C4) (Cruz Biotechnology, Inc.) and visualized by ECL immunoblot detection kit (Amersham Life Science).

### 2.6 Analysis of bicistronic SFV vector cytotoxicity in BHK-21 cells

Cytotoxicity of SFV vectors was analyzed as described by Garmashova et al., 2006. Replicon RNA was obtained from pSFV-PL-Pac, pSFV-Pac-EMCV-bcl2, pSFV-Pac-CR-bcl2 and pSFV-Pac-PR-bcl2. 10^6^ BHK-21 cells were electroporated with 5 μg of RNA. Cells were seeded into wells (growth area 2.0 cm^2^ per well; Cellstar, Greiner bio-one plates) and selected with puromycin (10 μg/ml) from 6 h post-transfection. Viable adherent cells were determined at 6, 24, 48 and 72 h post-transfection using Trypan blue (Flow Laboratories).

The viability of infected cells was also analyzed by WST-1 assay (Roche). The assay is based on the reduction of WST-1 to a water-soluble formazan dye by viable cells. BHK-21 cells were seeded in 96-well plates (7 × 10^3^ cells/well), grown for 18 h and infected with VLPs containing recombinant replicons at moi = 10. Control cells were mock-infected. Infected cells were analyzed 6, 24 or 48 h post-infection (p.i.) by adding 10 μl of WST-1 to each well, followed by incubating the plate for 1 h and measuring the change in color intensity at 450 nm in a microplate reader.

### 2.7 Immunofluorescence microscopy

Cells were grown on cover slips and infected for selected times, mock-infected cells were used as controls. Cells were washed with PBS, fixed with 4% paraformaldehyde for 10 min at room temperature and permeabilized with cold methanol for 7 min at −20 °C. Cells were then washed with PBS, blocked in the 3% BSA-PBS and incubated for 1 h with primary antibody (mouse anti-cytochrome *c* monoclonal antibody (BD Pharmingen) or rabbit polyclonal antibody against SFV nsP1). Then the cells were washed again with PBS and incubated with a AlexaFluor 568 (Invitrogen) or Cy3 conjugated secondary antibody for 1 h, washed three times with PBS and air-dried. Staurosporine (final concentration 0.5 μM) was added to mock-infected cells 1–2 h before fixing to induce release of cytochrome *c* from mitochondria. Samples were analyzed on an Olympus U-RFL-TX microscope or a Bio-Rad MRC-1024 confocal microscope.

### 2.8 Analysis of the viability of AT3 cells infected with SFV VLPs

3 × 10^6^ AT3-neo or AT3-bcl2 cells were infected with VLPs in serum-free RPMI1640 media supplemented with 0.2% BSA. The amount of VLPs used for infections corresponded to a moi = 20 for BHK-21 cells. At 12 h p.i., EGFP positive cells were separated using a BD FACSAria cell sorter; EGFP-positive cells were seeded in 24-well plates. Viable cells were determined at 24 and 48 h p.i. using Trypan blue (Flow Laboratories).

## 3 Results

### 3.1 Expression of d1EGFP and Bcl-2 using bicistronic SFV vectors

**Fig. 2:**
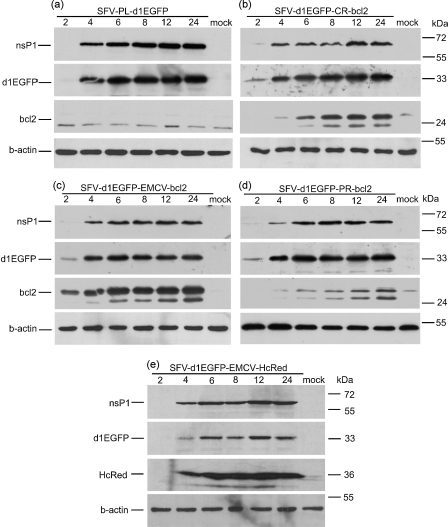
Expression of SFV nsP1, d1EGFP, Bcl-2 and HcRed proteins in infected BHK-21 cells at 2, 4, 6, 8, 12 and 24 h p.i. Infected cells (9 × 10^5^ cells/well) were collected, lysed and equal amounts of lysates analyzed by immunoblotting; beta-actin was used for normalization. Lysates of cells infected with SFV-PL-d1EGFP, SFV-d1EGFP-CR-bcl2, SFV-d1EGFP-EMCV-bcl2, SFV-d1EGFP-PR-bcl2 and SFV-d1EGFP-EMCV-HcRed VLPs are shown in panels a–e, respectively. Time of infection is shown at the top of each lane. Mock—mock-infected BHK-21 cells; lines at the left indicate positions of corresponding proteins; lanes at the right indicate positions of molecular mass standards. Experiments were repeated twice, with similar results.

To study the expression of foreign proteins using the bicistronic replicons, BHK-21 cells were infected with SFV-d1EGFP-CR-bcl2, SFV-d1EGFP-EMCV-bcl2 and SFV-d1EGFP-PR-bcl2 VLPs and in addition, with monocistronic SFV-PL-d1EGFP VLPs. Samples were collected at 2, 4, 6, 8, 12 and 24 h p.i. and analyzed by immunobloting. Expression of SFV nsP1 was generally detectable by 2 h p.i. and increased up to 8–12 h p.i. (Fig. 2a–e). The amount of nsP1 was approximately equal for all bicistronic vectors (Fig. 2b–d). Expression of d1EGFP was also detectable by 2-4 h post-infection (Fig. 2a–d) and was highest in SFV-PL-d1EGFP infected cells (Fig. 2a). Major differences were observed with Bcl-2 expression, which was detected on immunoblots as two bands (Fig. 2b–d). Bcl-2 expression was strongest and earliest (2 h p.i.) in SFV-d1EGFP-EMCV-bcl2 infected cells (Fig. 2c). In cells infected with SFV-d1EGFP-CR-bcl2 (Fig. 2b), expression of Bcl-2 was detected 4 h p.i. Expression of Bcl-2 was also found in cells infected with SFV-d1EGFP-PR-bcl2 (Fig. 2d) although levels were lower compared to IRES containing constructs.

To assess the ability of the bicistronic vectors to express proteins in general, Bcl-2 sequences were replaced with non-cytotoxic HcRed. The resulting replicons SFV-d1EGFP-EMCV-HcRed, SFV-d1EGFP-CR-HcRed and pSFV-d1EGFP-PR-HcRed were used to infect BHK-21 cells. There was no significant difference in the expression of nsP1 and d1EGFP proteins between bicistronic vectors also expressing HcRed or Bcl-2 (Fig. 2e for SFV-d1EGFP-EMCV-HcRed, data not shown for SFV-d1EGFP-CR-HcRed and pSFV-d1EGFP-PR-HcRed). Expression of HcRed was strongest for SFV-d1EGFP-EMCV-HcRed (detectable by Western blotting by 4 h p.i.; Fig. 2e). This is in agreement with previous observations suggesting that the highest expression levels of the second target proteins are achieved with SFV-d1EGFP-EMCV vectors. The apparent delay in detection of protein expression (2 h p.i. for Bcl-2 versus 4 h p.i. for HcRed) is most likely due to the quality of the HcRed antibody (Clontech).

Thus, vectors containing IRES elements expressed Bcl-2 earlier and to higher levels. Presence of a duplicated subgenomic promoter or IRES element following the Bcl-2 encoding sequence did not have any major effect on the time-course of infection, however, differences in expression levels of the first marker protein, d1EGFP, were observed.

### 3.2 Effects of infections by recombinant SFV VLPs on host cell protein synthesis

**Fig. 3:**
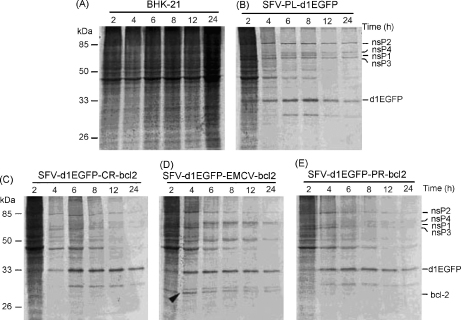
Effects of VLP infection on the host cell protein synthesis. BHK-21 cells (9 × 10^5^ cells/well) either mock-infected (A) or infected with SFV-PL-d1EGFP (B), SFV-d1EGFP-CR-bcl2 (C), SFV-d1EGFP-EMCV-bcl2 (D) or SFV-d1EGFP-PR-bcl2 (E) VLPs were metabolically labelled at 2, 4, 6, 8, 12 and 24 h p.i. Equal amounts of collected cell lysates were analyzed by SDS-PAGE, followed by autoradiography. Time of infection is shown at the top of each lane. Positions of molecular mass standards are indicated on the left, positions of SFV nsP1–nsP4, d1EGFP and Bcl-2 are indicated at the right. Arrow on panel D positions Bcl-2 expressed by SFV-d1EGFP-EMCV-bcl2.

Metabolic labelling was used to study the effects of infection by SFV VLPs on host cell protein synthesis. Infected BHK-21 cells were pulse labelled at 2, 4, 6, 8, 12 and 24 h p.i. In contrast to mock-infected cells, where protein synthesis is ongoing (Fig. 3A), translation in infected cells was rapidly inhibited (Fig. 3B–E). Shutdown of host protein synthesis was detected as early as 4 h p.i. (Fig. 3B–E). High-level and continuous expression of Bcl-2 was visible in cells infected with SFV-d1EGFP-EMCV-bcl2 (Fig. 3D). In cells infected with other bicistronic vectors, expression of Bcl-2 could only be detected by Western blot (Fig. 2b–d). These results indicate that high-level, continuous expression of Bcl-2 from a bicistronic replicon vector does not protect against shut-off of host cell protein synthesis.

### 3.3 Survival of cells infected with Bcl-2 expressing bicistronic replicons

It has been shown that similar to wild-type virus, alphavirus based replicon vectors induce apoptotic death of infected vertebrate cells (Glasgow et al., 1997, 1998; Scallan et al., 1997) and that over-expression of Bcl-2 can protect cells against alphavirus-induced apoptosis (Lundstrom et al., 1997; Scallan et al., 1997).

**Fig. 4:**
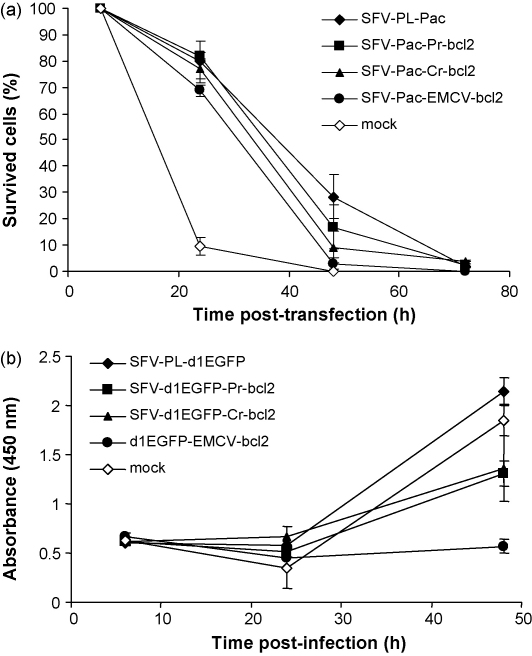
Analysis of survival (a) and viability (b) of cells infected with SFV replicons expressing Bcl-2. (a) Percentages of viable adherent BHK-21 cells at 6, 24, 48 and 72 h p.i. are shown. Puromycin selection was applied at the 6 h post-transfection. (b) Measuring cell viability by WST-1 assay at 6, 24 and 48 h p.i. Absorbance at 450 nm was measured 1 h after adding WST-1 reagent. Error bars represent standard deviation.

To study the effect of Bcl-2 expression from bicistronic vectors on cell death, BHK-21 cells were transfected with SFV-PL-Pac, SFV-Pac-EMCV-bcl2, SFV-Pac-CR-bcl2 and SFV-Pac-PR-bcl2 replicons or mock-transfected, and puromycin selection was applied 6 h post-transfection. Almost all mock-transfected cells died within 24 h of adding puromycin (Fig. 4a). The transfected cells survived longer and the percentage of viable adherent cells was found to be similar at the selected time points (Fig. 4a). Most of the transfected cells were dead 3 days post-transfection regardless of the replicon. Thus, our results demonstrate that high-level expression of Bcl-2 does not protect BHK-21 cells against SFV induced death. Similar results were also obtained when Annexin-V PE conjugate or propidium iodide were used to label apoptotic and necrotic cells (data not shown).

The WST-1 assay, used to analyze cell viability, measures mitochondrial activity in cells. Results obtained with this assay were similar to cell survival experiments: expression of Bcl-2 from bicistronic vectors (SFV-d1EGFP-CR-bcl2, SFV-d1EGFP-EMCV-bcl2 or SFV-d1EGFP-PR-bcl2) did not provide any protective effect (Fig. 4b). In fact, viability of cells infected with bicistronic replicons at 48 h p.i. was reduced, compared to cells infected with SFV-PL-d1EGFP (Fig. 4b). Infection with SFV-d1EGFP-EMCV-bcl2 caused the most important reduction in cell viability, which is in accordance with our previous data. (Fig. 4a). This tendency was also seen when cells infected with SFV-d1EGFP-EMCV-HcRed, SFV-d1EGFP-CR-HcRed and SFV-d1EGFP-PR-HcRed VLPs were analyzed by WST-1 assay (data not shown), indicating that not Bcl-2 expression, but rather the second gene expression unit (especially EMCV-IRES) is responsible for this effect. Taken together, our results suggest that Bcl-2 expression does not have a detectable effect on the viability of SFV-infected BHK-21 cells.

### 3.4 SFV infection does not induce release of cytochrome *c* from mitochondria

**Fig. 5:**
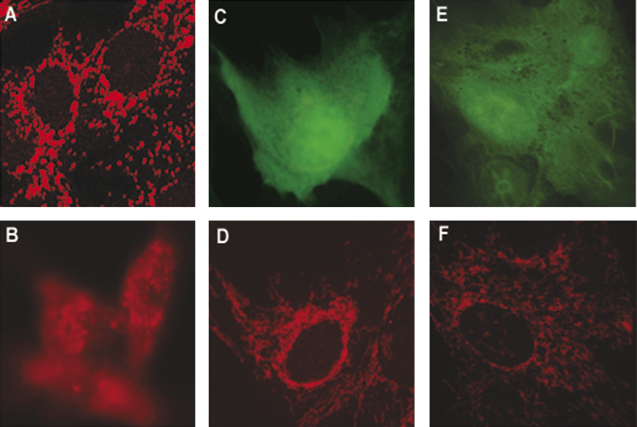
Sub-cellular localization of cytochrome *c* in BHK-21 cells. (A) Localization of cytochrome *c* in mock-infected BHK-21 cells or (B) in mock-infected BHK-21 cells treated with staurosporine. BHK-21 cells infected with SFV-PL-d1EGFP VLPs were visualized at 4 h p.i. by d1EGFP fluorescence (C) and localization of cytochrome *c* was analyzed (D). d1EGFP fluorescence and cytochrome *c* localization 24 h p.i are shown on panels E and F, respectively.

The anti-apoptotic effect of Bcl-2 is connected to its ability to prevent the release of cytochrome *c* from mitochondria (Kluck et al., 1997; Yang et al., 1997). Apoptosis induced by death receptors or by ER stress bypasses this mitochondrial pathway and is, therefore, relatively insensitive to protection by Bcl-2 (Scaffidi et al., 1998). The finding that high-levels of Bcl-2 expression did not have a protective effect against SFV induced death suggests that apoptosis in SFV-infected BHK-21 cells may not involve the mitochondrial pathway. To determine whether the mitochondrial pathway is activated in SFV-infected BHK cells, localization of cytochrome *c* was visualized by immunofluorescence in BHK-21 cells infected with the SFV-PL-d1EGFP 4 and 24 h p.i. Localization of cytochrome *c* in mock-infected BHK-21 cells was mitochondrial as evidenced by dotty localization (Fig. 5A). In cells, treated with the non-selective protein kinase inhibitor staurosporine, cytochrome *c* staining was diffuse, demonstrating its release from mitochondria (Fig. 5B). In cells infected with SFV-PL-d1EGFP, the cytochrome *c* staining maintained a granular pattern 4 h p.i. (Fig. 5C and D), indicating that when shut-off of cellular gene expression starts cytochrome *c* was not released from mitochondria. Furthermore, the cytochrome *c* localization pattern was unchanged even at 24 h p.i. (Fig. 5E and F). We conclude that induction of cell death in SFV replicon-infected BHK-21 cells does not involve the release of mitochondrial cytochrome *c*. This result is consistent with the absence of a protective effect of Bcl-2.

**Fig. 6:**
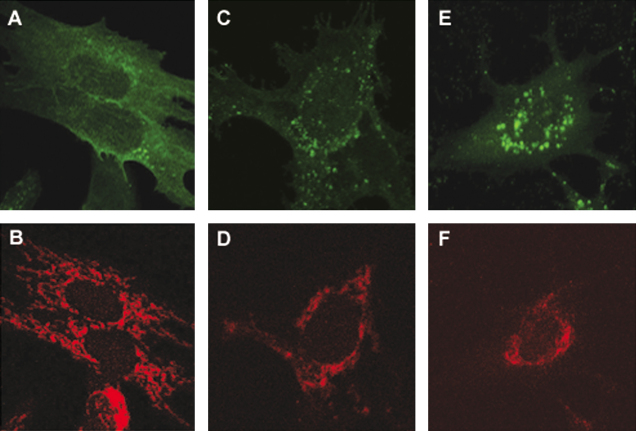
Sub-cellular localization of cytochrome *c* in BHK-21 cells infected with SFV4 virus particles (moi = 1). (A, C, E) nsP1 protein localization 4, 12 and 24 h p.i., respectively. (B, D, F) cytochrome *c* localization 4, 12 and 24 h p.i., respectively.

SFV replicons lack the coding sequences for structural proteins. To analyze the role of the structural proteins in the release of cytochrome *c* from mitochondria, BHK-21 cells were infected with SFV4 (moi = 1) and localization of cytochrome *c* was determined at 4, 12 and 24 h p.i. By 4 h p.i. infected cells did not release cytochrome *c* (Fig. 6A and B). At 12 and 24 h p.i. detection of cytochrome *c* became more difficult due to extensive cytopathic effects. This includes cell rounding and re-localization of mitochondria into the perinuclear region (Fig. 6C–F); all these effects were more extensive than in SFV-PL-d1EGFP infected cells (Fig. 5). Nevertheless, the localization pattern of cytochrome *c* remained dotty indicating that its mitochondrial localization was preserved (Fig. 6C–F). Taken together, these results indicate that for both replicon vector and SFV4 infection, BHK-21 cells do not release cytochrome *c* from the mitochondria.

### 3.5 Bcl-2 expression does not protect SFV-infected AT3-neo cells against cell death

To analyze whether the absence of cytochrome *c* release and lack of the protection against SFV induced cell death are specific to BHK-21 cells, two lines of AT3 cells, AT3-neo (Cepko et al., 1984) and AT3-Bcl2 (Levine et al., 1993) cells were also characterized.

**Fig. 7:**
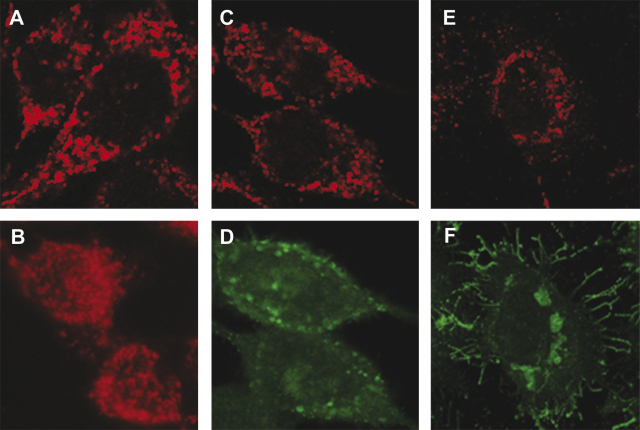
Sub-cellular localization of cytochrome *c* in AT3-neo cells. Localization of cytochrome *c* in mock-infected AT3-neo cells or in mock-infected AT3-neo cells treated with staurosporine is shown on panels A and B, respectively. Localization of cytochrome *c* in AT3-neo cells infected with SFV4 (moi = 1) 4 and 24 h p.i. is shown on panels C and E, respectively. Localization of nsP1 at 4 and 24 h p.i. is shown in panels D and F, respectively.

**Fig. 8:**
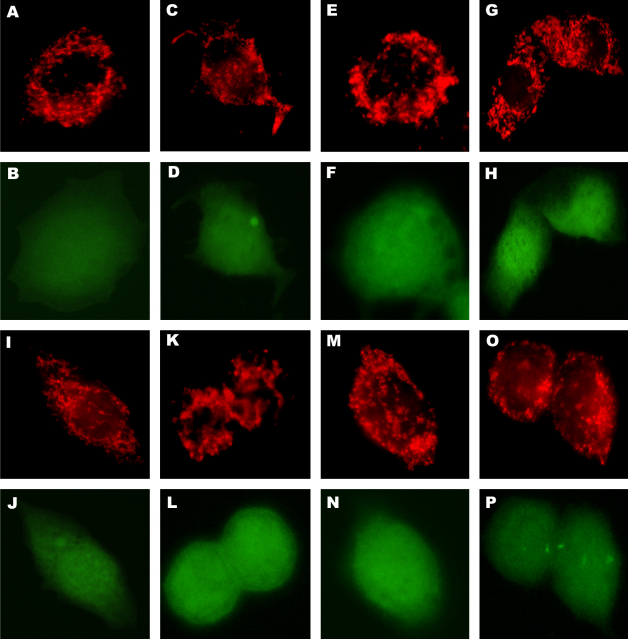
Sub-cellular localization of cytochrome *c* in AT3-neo (panels A–H) and AT3-bcl2 cells (panels I–P). Cells were infected with SFV-d1EGFP-EMCV-bcl2 VLPs (panels A–D and I–L) or SFV-PL-d1EGFP VLPs (panels E–H and M–P) and analyzed either 6 h p.i. (panels A, B, E, F, I, J, M and N) or 24 h p.i. (panels C, D, G, H, K, L, O and P). d1EGFP (green) was detected by auto-fluorescence and cytochrome *c* (red) by indirect immunofluorescence.

Firstly, AT3-neo cells were infected with SFV4 and cytochrome *c* distribution was determined. Infected AT3-neo cells showed extensive virus-induced cytopathic effects but as with BHK-21 cells no detectable release of cytochrome *c* (Fig. 7). Similarly, no detectable release of cytochrome *c* was observed in AT3-neo or AT3-bcl2 cells infected with SFV-d1EGFP-EMCV-bcl2 (Fig. 8A–D and I–L) or SFV-PL-d1EGFP VLPs (Fig. 8E–H and M–P). Therefore, we conclude that the inability of SFV to induce cytochrome *c* release from mitochondria was not a BHK-21 cell line-restricted phenomenon.

**Fig. 9:**
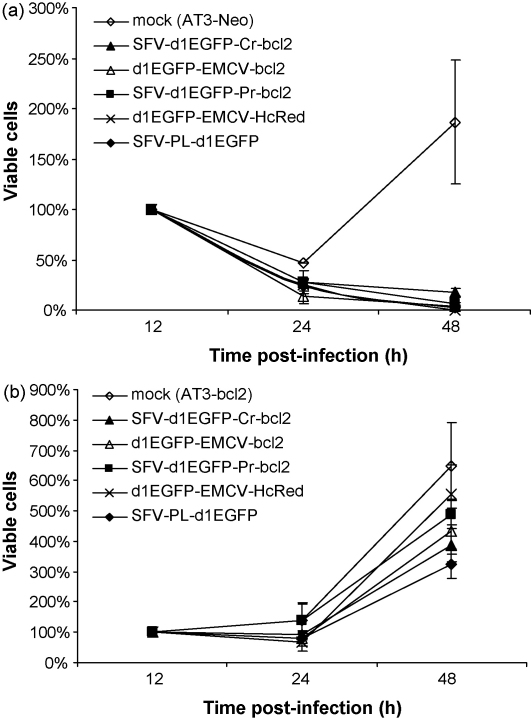
Analysing survival of AT3-neo (a) and AT3-bcl2 (b) cells infected with SFV replicons expressing Bcl-2 or HcRed. Number of viable cells at 12 h p.i. (sorted on a BD FACSAria cell sorter) was taken as 100%; numbers of viable cells at 24 and 48 h p.i. are shown. Error bars represent standard deviation.

Secondly, the viability of the AT3-neo and AT3-bcl2 cells, infected with SFV VLPs was analyzed. It should be noted that we were unable to obtain highly efficient infection of the AT3-neo and especially the AT3-bcl2 cells by use of the SFV VLPs. Typically, up to 20% of AT3-neo and 10% of AT3-bcl2 cells were infected when the amount of VLPs, corresponding to moi = 20 in BHK-21 cells, was used for infection; using larger amounts of VLPs did not result in higher percentages of infected cells. Thus, we conclude that AT3 cells are not as susceptible for SFV infection as BHK-21 cells and sorting of EGFP positive (i.e. infected) cells before the viability analysis was required. This revealed that, in the case of AT3-neo cells, expression of Bcl-2 by any SFV replicon vector did not provide any protective effect and almost all infected cells were dead by 48 h p.i. (Fig. 9a). Thus, the results were highly similar to those obtained in BHK-21 cells. In contrast, we found that almost all AT3-bcl2 cells, infected with SFV VLPs, were viable at 24 h p.i. and over the next 24 h numbers of viable cells rapidly increased in all samples. This indicates efficient cell division (Fig. 9b). Since cell sorting ruled out the possibility of non-infected cells being present in analyzed samples, we concluded that AT3-bcl2 cells, infected with SFV replicons were either able to recover from infection or, alternatively, established a persistent infection. Our observation that surviving AT3-bcl2 cells rapidly lost EGFP fluorescence favours the first option. Expression of Bcl-2 by SFV replicons had little or no effect on survival and subsequent division of infected AT3-bcl2 cells. All results taken together we conclude that not bcl-2 expression as such but some other property of AT3-bcl2 cells is likely responsible for this phenomenon.

## 4 Discussion

Previously published data on mechanisms of alphavirus-induced cell death are controversial (Glasgow et al., 1997; Griffin, 2005; Li and Stollar, 2004). On the one hand, induction of apoptotic cell death via the death receptor pathway has been suggested (Li and Stollar, 2004; Nava et al., 1998); on the other hand, it has been shown that over-expression of the anti-apoptotic Bcl-2 can protect against alphavirus-induced apoptosis and pathogenesis (Levine et al., 1993; Lundstrom et al., 1997; Mastrangelo et al., 2000; Scallan et al., 1997). However, in these studies different alphaviruses, strains, cell lines and experimental systems were used. It is possible that mechanisms, by which cell death is induced may differ between viruses, cells etc.

In this study, shut-off of cellular translation and cell death in SFV-infected BHK-21 cells were studied using a novel bicistronic replicon vector system. This avoids transient or stable expression of Bcl-2 in host cells. In transiently transfected cells, cell metabolism can be seriously altered by the transfection procedure (Lepik et al., 2003). Stable expression of Bcl-2, known to have oncogenic properties (Reed, 1994; Reed et al., 1991), may lead to adaptation of the cell, which in turn might affect virus replication and the outcome of infection. It is also important to mention that both transient and stable cellular expression are mediated by cellular RNA polymerase II and are, therefore, subjected to the alphavirus-induced transcriptional shutdown, which starts early in infection.

In this study, Bcl-2 was expressed using novel vectors containing IRES elements. The use of IRESs has significant advantages over other systems. The first important advantage, as shown by our results (Fig. 2), is that IRES-mediated expression of Bcl-2 can take place directly from incoming genomic RNA. In contrast, expression from the subgenomic promoter requires replication and is activated later on in infection. Early expression of Bcl-2 may be crucial since it is not known when and how cell death is induced. It is possible that expression of Bcl-2 from the subgenomic promoter is too late to protect from cell death. Another important advantage of IRES-mediated expression is the level of expression, which is significantly higher than from the minimal subgenomic promoter (Fig. 2). It is also important to mention that at least for the EMCV-IRES, synthesis of Bcl-2 was resistant to inhibition of cellular translation (Fig. 3D).

Detection of Bcl-2 protein by immunoblotting in BHK-21 cells infected with VLPs of SFV1 vectors harboring *bcl-2* revealed a lower molecular band, which increased in intensity proportional to Bcl-2 expression. This is presumably protein expressed from an internal initiation site (Tsujimoto and Croce, 1986). The truncated protein could also be the product of Bcl-2 cleavage by endogenous caspase-3, resulting in curtailed Bcl-2 with proapoptotic activity (Cheng et al., 1997; Kirsch et al., 1999). The latter localizes to mitochondria and causes the release of cytochrome *c*, thus, promoting further caspase activation as a part of a positive feedback loop (Kirsch et al., 1999). It has been shown that alphaviruses induce apoptosis in Bcl-2-overexpressing cells by caspase-mediated proteolytic inactivation of Bcl-2 (Grandgirard et al., 1998). Viral capsid protein was suggested to trigger activation of the cell death machinery. However, in this study neither release of cytochrome *c* nor increase in the proportion of truncated product due to feedback loop action was observed. Infection of cells with VLPs eliminated the possibility that capsid protein alone triggers apoptosis.

Simultaneous expression of two target proteins was achieved with all the bicistronic SFV expression vectors constructed in this study. The expression level of d1EGFP was slightly reduced for bicistronic vectors, compared to the monocistronic SFV-PL-d1EGFP, independent of the nature of the second gene (*bcl-2* or *HcRed*) (Fig. 2). Interference between the native subgenomic promoter and the duplicated promoter or IRES element may have influenced expression from the first promoter. It is also possible that presence of the IRES elements had some effect on replication. However, infectivity of bicistronic vectors was not altered. Taken together, these results indicate that different IRES elements can be used to construct novel and efficient bicistronic SFV vectors.

In this study, we show that early and high-level of Bcl-2 expression, achieved with bicistronic SFV replicon vectors, did not have a detectable effect on host protein synthesis shut-off (Fig. 3) or cell death (Fig. 4). This cannot be attributed to the delayed expression of *bcl-2* since its high expression levels were observed at early time points (Fig. 2). These findings are coherent with our data suggesting that infection of BHK-21 cells with SFV replicons or with SFV4 does not cause the release of cytochrome *c* from mitochondria (Figs. 5 and 6). This effect was not restricted to BHK-21 cells since similar results were also obtained for AT3-neo (Figs. 7 and 8) and AT3-bcl2 (Fig. 8) cells. The release of cytochrome *c* in SFV-infected BHK-21 or AT3-neo cells did not even occur at late time points. This explains the lack of protective effect by Bcl-2 expression against cell death in infected cells, as observed by us.

At the same time, the mechanism of cell death in infected cells remains unknown. We can rule out that binding of virions to the cellular receptor and/or virus internalisation have crucial roles, since induction of cell death does not depend on whether the cells are infected with virus or transfected by infectious transcripts. It seems most likely that cell death is induced by some non-mitochondrial pathway. This is consistent with previous data, which suggested that apoptosis in alphavirus infected cells uses the death receptor pathway (Nava et al., 1998; Li and Stollar, 2004). However, our findings do not exclude the possibility that cell death in SFV-infected BHK-21 cells may be triggered by the ER pathway.

A delay of cell death in SFV-infected AT3 cell line expressing *bcl-*2 has been reported (Scallan et al., 1997). Our results confirm that the AT3-bcl2 cell line is remarkably resistant to infection by SFV VLPs. However, our data is more coherent with the hypothesis that these cells are not just delaying cell death but are also capable to recover from SFV infection. The finding that Bcl-2 expression by SFV replicons did not have any effect on the survival of AT3-neo cells suggests that resistance of the AT3-bcl2 cells to the SFV infection most likely represents an indirect effect of Bcl-2. The *Bcl-2* gene is potent oncogene and its constitutive expression significantly changes cell cycle and gene expression. It is indeed evident from data presented in Fig. 9 that AT3-bcl2 cells grow much more rapidly than AT3-neo cells. It could be hypothesized that constitutive expression of Bcl-2 in AT3-bcl2 cells may have resulted in changes protecting cells from virus infection. The most likely candidates for roles of these factors may be the components of the innate immune system and/or host factor(s) like rat zinc-finger antiviral protein, which provides resistance against alphavirus infection (Bick et al., 2003).
